# Digital Health Technology to Support Health Care Professionals and Family Caregivers Caring for Patients With Cognitive Impairment: Scoping Review

**DOI:** 10.2196/40330

**Published:** 2023-01-11

**Authors:** Mohamed-Amine Choukou, Funminiyi Olatoye, Reg Urbanowski, Maurizio Caon, Caroline Monnin

**Affiliations:** 1 Department of Occupational Therapy College of Rehabilitation Sciences Rady Faculty of Health Sciences, University of Manitoba Winnipeg, MB Canada; 2 Centre on Aging, University of Manitoba Winnipeg, MB Canada; 3 School of Management of Fribourg, University of Applied Sciences and Arts Western Switzerland (HES-SO) Fribourg Switzerland; 4 Neil John Maclean Health Sciences Library, University of Manitoba Winnipeg, MB Canada

**Keywords:** digital health, behavior change, mental health, cognitive impairment

## Abstract

**Background:**

Digital health technology is a promising way of supporting health care providers and family caregivers as they care for patients with cognitive impairment.

**Objective:**

This scoping review aimed to portray the use of digital health technology to assist health care providers and family caregivers in caring for patients with cognitive impairment who live in the community or in a facility.

**Methods:**

We conducted a scoping review of peer-reviewed scientific articles available in MEDLINE, PsycINFO, Scopus, and CINAHL with Full Text, as well as gray literature available in preprint servers, theses depositories, and various national and international dementia organizations’ websites. The search yielded 975 articles, of which we included 7 (0.7%) in the review.

**Results:**

Of the 7 interventions included in the retrieved manuscripts, 2 (29%) were digital calendar reminder systems to support activities of daily living and medication management; 2 (29%) were apps on tablet devices to simulate the presence of family before therapy interventions; 1 (14%) was a social robot used in therapeutic sessions to include elements of musicotherapy, reminiscence, cognitive games, and relaxation; 1 (14%) was a commercially available computer system that provides access to various recreational leisure activities; and 1 (14%) was a web-based self-management support system that helps family caregivers to deal with behavior changes in a relative with dementia. Of the 7 articles, only 1 (14%) reported on the use of a behavior change theory, namely a comprehensive process model of engagement coupled with cognitive stimulation therapy.

**Conclusions:**

Literature on the topic is scarce, recent, and heterogeneous. There is a clear need for a theoretical framework to conceptualize and govern the use of behavior change models that incorporate technology for patients with cognitive impairment.

## Introduction

### Background

The aging of the population will intensify in the coming decades, increasing the prevalence of disease and disability, in particular, the impairment of cognitive functions [1]. In Canada, for example, approximately 1 in 4 seniors aged ≥85 years have been diagnosed with dementia [2]. The growing number of people with cognitive impairment will result in high demands on health care systems. Cognitive impairment reduces the quality of life of older adults and increases the risk of dementia and mortality [3,4]. In this paper, the term *cognitive impairment* refers to the various etiologies known to characterize cognitive impairment, such as vascular conditions, neurological conditions, and stroke. The ability of people with cognitive impairment to retain information, learn new things, or concentrate is impaired, leading to a decrease in autonomy and independence and poor social participation. Cognitive impairment affects the patients and those around them, particularly family caregivers and the health care professionals providing care to alleviate the condition [5,6]. Family caregivers’ activities require a constant connection to the person with cognitive impairment and the activities they require to perform to function and live safely (eg, maintain a career, home, and family life). The burden experienced by people taking care of a person with cognitive impairment is associated with an adverse emotional state and can be more substantial in terms of physical sequelae and financial and social consequences [7].

Responsible health and social systems should support caregivers to help maintain persons with cognitive impairment in their homes for as long as possible. From the health care provider’s perspective, the delivery of quality patient-oriented care seems to be determined by their ability to understand the particular care and heterogeneous communication needs of people with cognitive impairment [8,9]. Digital health technology is a promising way of supporting patients with cognitive impairment in that technology can be an enabler of behavior change in patients, family caregivers, and health care professionals. One could imagine how technology may foster communication among users (patients, caregivers, and care providers) and affect daily routines among all stakeholders. This paper examines the use of digital health technology as a plausible way of supporting health care providers and family caregivers as they care for people with cognitive impairment. According to the US Food and Drug Administration, digital health technologies “use computing platforms, connectivity, software, and sensors for health care and related uses” [10]. Digital health technology has been deemed valuable and efficient in supporting health care providers and family caregivers in different populations, such as children with special health care needs [11], patients undergoing hematopoietic cell transplantation [12], and patients with Parkinson disease [13], as well as patients requiring cardiovascular care [14], pulmonary rehabilitation [15,16], mental health rehabilitation [17], and cognitive rehabilitation [18]. Digital health technology faces challenges at individual and system levels, such as lack of presence and in-person contact [19] as well as ethics and data governance [20]. The design of a digital health technology for patients with cognitive impairment is itself challenging because of their complex needs and because they may be in no condition to engage in the design processes. Creative technologists should be aware of the importance of actively including the end users in the design process [21] and that the involvement of end users with cognitive impairment can be a complex task [22], which is even more critical when older adults are the end users [23]. When designing a digital health technology that persons with cognitive impairment will use, the task is cumbersome for many reasons related to awareness about their health needs, knowledge of potential ideas based on technology, and level of technology literacy.

In practice, digital health technology is understudied in people with cognitive impairment, family caregivers, and health care providers. This paper focuses on information and communication technologies deployed with or without additional digital technologies to look more closely at digital health technology that claims to support health care professionals and family caregivers caring for patients with cognitive impairment by providing them with digital support and a sense of presence using technologies such as, but not limited to, anthropomorphic agents, digital tools, robots, apps, and multimedia systems. The typical intervention sought in this review would be based on technology that allows digital dyadic communication between patients with cognitive impairment and their health care providers and family caregivers.

### Objectives

In this paper, we aimed to depict the use of digital health technology to assist health care professionals and family caregivers in caring for patients with cognitive impairment who live in the community or in a facility. Recent literature portraying available technology applications to support the informal caregiver of a person with cognitive impairment showed the impact of technology in reducing the caregiver’s burden and the patient’s social isolation [24]. According to the literature, social support is still needed to improve the adherence and effectiveness of digital health technology–enabled telerehabilitation [25]. Although studies show an impact of technology on occupational performance and quality of life in community and health care settings [26,27], more research on the impact of digital health technology settings [26,27] and users’ existing knowledge and informational needs [25,27] as well as feasibility and acceptability [25] is needed. Furthermore, Bell et al [28] advocate for including motivational frameworks and behavior change interventions in digital health intervention development, increasing service engagement in young people and lived experience involvement in digital intervention development. The objectives of this paper were to (1) identify which digital health interventions were tested in patients with cognitive impairment, health care professionals, and family caregivers, as well as the associated behavior change strategies; (2) identify the behavior change theories involved in the included literature that relates to patients, health care providers, and family members; (3) provide an overview of the effects of these interventions on patients with cognitive impairment, health care professionals, and family caregivers; and (4) identify and comprehend the benefits, challenges, and influencers of using the digital health technology to assist health care professionals and family members caring for patients with cognitive impairment.

## Methods

### Design

We performed a scoping review of peer-reviewed scientific articles following the recommendations of Arksey and O’Malley [24] and Levac et al [25]. We followed the PRISMA-ScR (Preferred Reporting Items for Systematic Reviews and Meta-Analyses extension for Scoping Reviews) guidelines [26]. Information was extracted from the included manuscripts using customized calibrated forms that were tested by the team before their use. Data charting was performed by 1 coauthor per paper and was validated by a second team member. There was no consultation exercise at the end of this scoping review.

### Search Strategy

The dementia and cognitive impairment search concept was adapted from the Cochrane Dementia and Cognitive Improvement group [27]. To develop the search strategy, a health sciences librarian (CM) consulted with the team (MAC, FO, RU, and MC). Using a combination of controlled vocabulary and keywords, the team developed search concepts for telepresence and behavior changes (Multimedia Appendix 1). The telepresence search concept incorporated Medical Subject Headings (MeSH) terms and keywords representative of robotics and telecommunications, including the MeSH terms *Artificial Intelligence*, *Human-Robot Interactions*, *Self-help Devices*, *Wearable Electronic Devices* and *Robotics*. The behavior changes concept included the MeSH terms *Health Behavior*, *Self-control*, *Self-examination*, and *Health Risk Behaviors*. In addition, we excluded articles indexed as child or infant only and limited them to English.

To locate gray literature, the librarian searched in preprint servers, theses depositories, and various national and international dementia organizations’ websites with the keywords *dementia* or *cognitive impairment and technology*. The search extraction took place in April 2021 in the following databases: MEDLINE (Ovid; 1946-2021), PsycINFO (Ovid; 1806-2021), Scopus (1970-2021), and CINAHL with Full Text (EBSCOhost; 1981-2021). All records were exported to EndNote (version X9; Clarivate), and duplicates were removed [29]. The screening process was completed in Rayyan software [30].

### Inclusion and Exclusion Criteria

To be included, the articles had to be written in English and present original qualitative or quantitative empirical data related to the use of a digital health technology (eg, a mobile app or a telepresence robot) to support health care delivery, family caregiving, and behavior change in patients with cognitive impairments. The articles had to refer to a part of, or an entire, behavior change approach and the related qualitative and quantitative instruments used to measure a patient’s behavior change, with reference to the behavior change technique taxonomy formulated by Michie et al [31]. The included articles had to be focused on adults with cognitive impairment (eg, dementia, traumatic brain injury, or stroke) and their health care providers and family caregivers. Search results reporting on surveys, technical development, perspectives, and literature reviews were excluded. Two independent reviewers performed the study selection. An article had to be judged relevant by both reviewers to be included. A third reviewer was consulted when the 2 first reviewers could not reach a consensus.

### Study Selection Process

**Figure 1:**
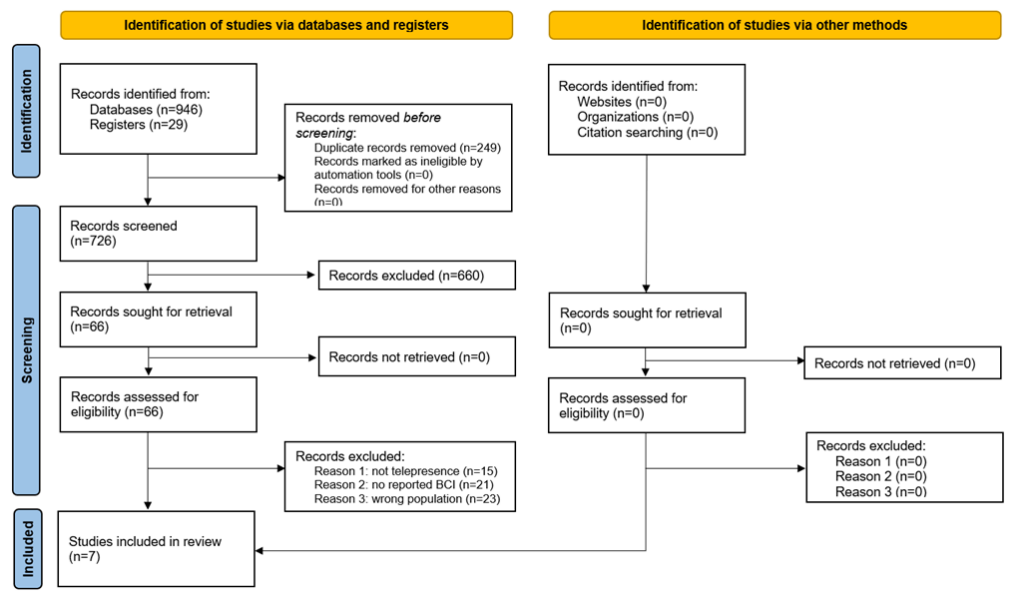
Study selection process.

The search yielded 975 articles, with 726 (74.5%) remaining after duplicates were removed (Figure 1). After screening titles and abstracts, of the 726 articles, 66 (9.1%) were retained and read in their entirety to determine eligibility. As a result, 89% (59/66) of the articles were rejected for various reasons (Figure 1). Thus, of the initial 975 articles, 7 (0.7%) were finally included in this scoping review.

### Data Extraction and Data Analysis

We gathered and charted the following information: the technology-enabled presence and telepresence interventions; technology used; profiles of the patients with cognitive impairment, family caregivers and health care professionals; outcome measures; intervention outcomes; and barriers to intervention implementation. Information about the factors affecting the success of digital health technology implementation was extracted from the discussion and limitations sections of the retrieved manuscripts and underwent thematic analysis. Two reviewers independently extracted the data, and all team members revised the extracted information and adjusted or completed the extraction if necessary. After a series of iterations and content validation, we charted the data and presented them in a tabular format in Microsoft Excel 2016.

## Results

### Characteristics of the Included Studies

The included studies were published between 2012 and 2020 in the United States (2/7, 29%), Canada (1/7, 14%), Mexico (1/7, 14%), Sweden (1/7, 14%), the Netherlands (1/7, 14%), and Japan (1/7, 14%). Of the 7 studies, 6 (86%) were peer-reviewed research articles, and 1 (14%) was a thesis presented to obtain the degree of doctor of occupational therapy [32]. Of the 7 manuscripts, 6 (86%) presented qualitative and quantitative information from small-scale empirical studies involving 2 to 18 participants in each profile studied (patients with cognitive impairment, health care providers, and family caregivers) [32-37]. The manuscripts centered around adults and older adults with Alzheimer disease, dementia, and Parkinson disease dementia living in various settings, such as geriatric residence, memory care unit, and home. Only 14% (1/7) of the studies presented data from a randomized controlled trial involving 81 family caregivers [38].

### Digital Health Interventions: Characteristics and Outcomes

Table 1 describes the included digital health interventions and their effects on patients with cognitive impairment, health care professionals, and family caregivers. As per the objective of the scoping review, the included manuscripts presented data from interventions targeting patients and health care providers (3/7, 43%) [33-35]; patients and family caregivers (1/7, 14%) [32]; patients with cognitive impairment, health care providers, and family caregivers (2/7, 29%) [36,37]; and family caregivers only (1/7, 14%) [38].

The included studies involved the following presence and telepresence interventions, all detailed in Table 1:

- Two digital calendar reminder systems to support activities of daily living [33] and medication management [36]
- Two apps on tablet devices to simulate the presence of family before therapy interventions [35] and to reduce agitations and support caregivers’ education [32]
- A social robot used in therapeutic sessions to include elements of musicotherapy, reminiscence, cognitive games, and relaxation [34]
- A commercially available computer system that provides access to various recreational leisure activities to encourage the engagement of people with dementia in activities and social interactions [37]
- A web-based self-management support system that helps family caregivers deal with behavior changes in a relative with dementia [38]

**Table 1:** Intervention description and effects on patients with cognitive impairment, health care professionals, and family caregivers.

Technology used	Intervention description	Patients	Health care professionals	Family caregivers	Outcome measure	Outcome	Reference number
Digital calendar with active reminders to support activities of daily living—description: RemindMe, a digital calendar developed to support people with cognitive impairment that includes three core functions: (1) scheduling of activities and customizing reminders in a user-friendly digital calendar, (2) active confirmation of reminders sent by SMS text message requiring users to acknowledge the prompt actively by responding to the SMS text message, and (3) a feedback system that registers information based on the user’s interaction; use: stand-alone	Patients received an individualized introduction, a written manual, and individual weekly conversations for 2 months with follow-up assessments after 2 and 4 months. All occupational therapists received training. The patients received rehabilitation as usual at the rehabilitation clinic together with structured support in the use of RemindMe as a support for memory and to plan and structure activities of daily living; setting: rehabilitation clinics	Eight patients (6 women) with a median age of 58 (range 26-68) years from 3 rehabilitation clinics in Sweden, who had cognitive impairment and needed support with planning, organizing, and remembering to perform activities of daily living	Seven female occupational therapists, specialists in rehabilitation for people with neurological impairments in primary care, with 2 to 25 years of experience; 5 had a bachelor’s degree in occupational therapy, and 2 had a bachelor’s degree with 1 year’s postgraduate education; all were experienced in using digital health technology for their patients, but no one was specialized in developing information and communication technologies	N/A^a^	Patient satisfaction (assessed with QUEST 2.0^b^) Field notes (patients’ opinions or interests or intentions) Logs in the calendar Interviews with occupational therapists (appropriateness, patient ability, implementability, and fit of RemindMe within the infrastructure) Data regarding the cost for patients to use RemindMe	Acceptability: +^c^ Demand: + Implementation: challenges Practicality: challenges Integration: +	[33]
Social robot Eva—description: an assistive anthropomorphic robot used in therapeutic sessions to include elements of musicotherapy, reminiscence, cognitive games (complete with wisdom sayings), and relaxation; use: stand-alone	Cognitive stimulation therapy that aims to actively mentally stimulate people with dementia through cognitive activities and reminiscence, multisensory stimulation, and group social contact. The cognitive stimulation therapy included a set of therapeutic sessions conducted by the robot Eva. The study was conducted in a lounge of the geriatric residence where all participants live; setting: nursing home	Nine people (6 women) with dementia living in a geriatric residence, aged between 74 and 95 (mean 83.77, SD 8.13) years	Six caregivers (4 women) participated in the study; they had an average of 3.2 years of experience as caregivers in a geriatric residence	N/A	Frequency and intensity of problematic behaviors (assessed with the NPI-NH^d^) Qualitative analysis	Decrease in frequency and intensity of problematic behaviors, agitation, depression, delusion, apathy, and irritability (–^e^) Behavior and mood change: cooperation, positive mood, less isolation, and positive facial and corporal expressions (+) Activities of daily living: feeding improvement, activity-level change, fewer naps, and participation in residence activities (+) Socialization: talkative, singing and laughing, and influence on other residents (+) Prevalence of the impact: fewer problematic behaviors, impact on caregivers’ burden, and sustained impact on behavior (+)	[34]
Tablet device (large screen size to accommodate potential visual impairment)—description: tablet-simulated presence therapy intervention; use: human enacted	Family caregivers asked to self-record a personalized 1-minute video based on how they would typically communicate with the study participant. Family videos are recorded either directly into the tablet device on the unit or at home using a personal device and later uploaded to the tablet device. Content tailored to help with particular challenges (eg, medication refusal and responsive [aggressive] behaviors); setting: hospital	Four hospitalized older adults (3 women) with dementia, aged 69 to 80 years. They had different types of diagnoses, including Alzheimer disease, vascular dementia, and Parkinson disease dementia	Two experienced full-time nurses who had basic training in dementia care. One had practiced 14 years in dementia care, and the other had 18 years of experience	N/A	Filmed footage and interviews	Patients: positive changes in the mood of all 4 older adults (+) How health care providers and health care professionals used the tablet device to interact with the patients significantly affected the outcomes. The care interaction among the patients required a person-centered approach. When a video delivered an effective message, it bridged the connection and helped older adults with dementia feel safe (+)	[35]
Tablet device—description: apps for reducing agitations and supporting caregivers’ education; use: human enacted	Tablet device–based program for family caregivers’ education and reducing problem behaviors in older adults with cognitive impairments. Four apps were used for the agitation-reduction component of the program. For the educational component, meetings or email correspondence with family caregivers occurred to discuss important information about dementia and how to interact with people with dementia; setting: care unit	Five older adults with dementia	N/A	Eight family caregivers received educational information; data were only collected from 6 of the participants	Participants’ scores on the Agitated Behavior Scale Interviews	Patients: a notable reduction in agitation during the use of the apps (–) Family caregivers: knowledge about specific aspects of dementia increased significantly after participating in the program (+)	[32]
A computer system designed for older adults in community settings—description: a commercially available system that encourages the engagement of people with dementia in activities and social interactions by providing access to various recreational leisure activities; use: human enacted	The system was made available for use, as part of a 6-month study, in a 26-apartment MCU^f^ for people with mild-to-severe dementia. The system was placed in the dining room of the MCU and was used there by health care professionals. It was wheeled into a side room or resident’s room by the researchers for individual sessions; setting: care unit	Five older adults residing in the MCU, aged ≥50 years	Seven health care professionals in the MCU, aged ≥18 years, interact directly with the individuals in the MCU	Four family caregivers of older adults living in the MCU, aged ≥18 years, who have visited their relative residing in the MCU at least monthly in the year preceding the study	Interviews with health care professionals and family caregivers	Health care professionals and family members reported benefits for residents, such as enjoyment, interactions and connections with others, and mental stimulation (+)	[37]
Medication reminder device—description: an automatic pill dispenser with audible and visual stimuli that remind users when to take their medication. When the alarm rings, the correct dose of medication is released into the lid opening. Users must then invert the device to obtain medication and stop the alarm; use: human enacted	If participants did not find any difficulty using the device, the device and its use were customized. Customized conditions included medication loaded into the device, loading schedule, location in the home, time of the alarm, and other individual considerations. The caregivers monitored device use during the first week of its use. They were asked to provide minimal assistance to users in using the devices (only when required); setting: home care ad community care	A total of 18 older adults (15 women) with dementia (mean age 81.2, SD 6.2, years), living at home, with a history of missed medication doses, overdoses, or need for verbal reminders to take medication once or more during a week	Eight caregivers and visiting nurses	Ten family members living separately	Self-administration medication rate is defined as the ratio of the number of doses taken independently to the number of all prescribed doses during 1 week Open questioning of users and their caregivers	Self-administration medication rate during 1 week showed improvement at 3 months (13/18 users; +) Reminder devices can improve medication adherence (+) Caregivers reported maintenance of normal blood pressure, reduction of caregivers’ burdens, and decreased care costs (+) Users reported gaining self-confidence and success at learning the skills necessary to use the device (+)	[36]
Web-based self-management support—description: web-based self-management support intervention to help family caregivers deal with behavior changes in a relative with dementia; email contact of a specialist dementia nurse and web-based videos and e-bulletins; use: human enacted	Family caregivers received 3 personal email contacts with a specialist dementia nurse (during 12 weeks). The nurse supported the family caregivers in managing behavior changes by giving feedback on assignments and tailoring support to the personal needs of the family caregivers. Other family caregivers received links to 6 web-based videos with assignments about different types of behavior changes, and they could choose how many videos they watched and assignments they completed. A third group of family caregivers received 6 e-bulletins containing practical information about different types of changes in behavior and how to manage them; setting: web based (caregiver support)	N/A	N/A	A total of 81 family caregivers (partner or relative) of people with dementia who live at home. They had contact with the person with dementia at least once a week	Primary outcome variable (self-efficacy) was measured using the Trust in Our Own Abilities instrument, a questionnaire in Dutch Presence and reaction scores for mood and behavior problems, measured by the Revised Memory And Behavioral Problem Checklist Occurrence of disruptive behavior and family caregivers’ reaction Dyadic Relationship Scale used to assess relationship between person with dementia and family caregiver	The web-based self-management support intervention involving email contacts did not lead to positive effects compared with web-based interventions without personal email contacts (–) The medium intervention involving web-based videos and e-bulletins showed no statistical improvements compared with the minor intervention involving e-bulletins only (no significant change)	[38]

^a^N/A: not applicable.

^b^QUEST 2.0: Quebec User Evaluation of Satisfaction With Assistive Technology, version 2.0.

^c^+: positive effect.

^d^NPI-NH: Neuropsychiatric Inventory, Nursing Home version.

^e^Negative effect.

^f^MCU: memory care unit.

Overall, using multiple methods and designs, the studies showed positive effects on patients with cognitive impairment, health care providers, and family caregivers (Table 1), without adverse effects on any digital health technology users. The studies reported some barriers faced by health care providers and family caregivers to implementing and using digital health technology.

### Behavior Change Theories Involved in the Included Literature

The study by Cruz-Sandoval et al [34] was the only one reporting on using a behavior change theory, which consisted of applying a comprehensive process model of engagement and cognitive stimulation therapy. None of the other studies explicitly applied a behavior change theory.

#### What Factors Influence Digital Health Technology’s Success in Assisting Health Care Professionals and Family Caregivers Caring for Patients With Cognitive Impairment?

Thematic analysis of the information extracted from the discussion and limitations sections of the included papers about the factors influencing digital health technology success revealed 18 factors. Table 2 summarizes these factors into 3 categories: benefits, challenges, and influencers of digital health technology success in each of the 3 groups: patients with cognitive impairment, health care professionals, and family caregivers.

**Table 2:** Benefits, challenges, and influencers of using digital health technology to support patients with cognitive impairment, health care professionals, and family caregivers.

	Benefits	Challenges	Influencers
Patient with cognitive impairment	Engagement in the activity Habit forming [33] Gaining self-esteem, motivation, and “having something to which to look forward” [37] Technology replaced or augmented activities [37]	Relevance and quality of the content Video content does not have to be older for residents to relate to it [37] Older content may be frustrating (“Residents were frustrated knowing they should recognize materials from a certain era but not being able to do so”) [37] Technical issues Technical issues and lack of user-friendliness of the system are frustrating for people with dementia [37] Improper operation of the device may be a reason for cessation [36] Patient-centered content Applications designed for the general population and ill-suited for patients with cognitive impairment [37] Gaming stations not suited to disease-related disabilities (eg, wheelchair vs access to station and station height) [37]	Planning and delivery of activities Whether the system was used one-on-one or in a group (intervention would probably be more beneficial in a one-on-one setting or small group) [37] Frequency of use [37]
Health care professional	Support and resources Equipped, trained, and having access to educational resources [32] The use of technology, specifically the iPad, throughout the program provided an easy and convenient way to deliver both interventions and education [32]	Workload Weekly telephone calls during a more extended period can become time consuming and problematic to implement in practice [33] Telephone calls might reveal other, new, problems, and therefore frequent telephone calls can be problematic to implement in daily practice [33] Lack of resources, especially time and personnel [37] Technology use The difficulty of keeping up to date with rapid technological development [33] Knowing how to use a tablet device and video applications [35] Not knowing how to use features or where applications were located [37] Limited previous experience in providing self-management support through email contacts [38] Attractiveness of content Boredom caused by limited content (too few episodes of television shows or movies) [37]	Involvement and professional development Involvement as a facilitator and motivator [37] Instruction and increased awareness of the importance of the integrated use of the various elements in an intervention [38] Adding information about the apps and creating case studies [32] Quality of content and convenience of technology Service accessed from various computers [37] Convenience and availability of information and media [37] Collaboration with families to improve care [35] (ie, building video content) Sustainability Long-term use: the health care professionals would have everything they need in 1 place for both education and intervention purposes [32] Cost Apps can be downloaded for little to no cost [32]
Family caregiver	Involvement Involvement leads to a better relationship [35]	Collaboration needed with a health care provider The family caregiver asked the occupational therapist for support in deciding about content building (what to include in videos) [35]	Involvement Involvement leads to a better relationship as well as safer and higher quality of care [35] Involvement as a facilitator and motivator [37] Caregiver’s help is a prerequisite for the use of the medication reminder device [36] Technology should be developed as a technical aid for use with family caregivers’ help [36] Resources and skills Family caregivers all had internet access and were often relatively young and well educated [38] Increased comfort with technology and finding information makes family caregivers more self-assured with accessing and using knowledge about dementia [32] Ease of use of the technology Adding information about the apps and creating case studies [32]

#### How Does Digital Health Technology Support Patients With Cognitive Impairment?

Although digital health technology encourages patients to participate in activities (videoconferencing and visualization of memories through photos and videos), they may encounter technical difficulties, find content less relevant than expected, and be less adapted to their conditions. The quality of activity planning seems to be a determinant of the success of digital health technology implementation.

#### How Does Digital Health Technology Support Health Care Professionals’ Work With Patients With Cognitive Impairment?

Although health care providers feel supported and resourceful when incorporating digital health technology into their interventions, they may face technical difficulties, find content less appealing than expected, and feel burdened by the increased workload. The success of digital health technology implementation seems to be influenced by health care providers’ active participation in the development and integrated use of the technology, collaboration with families, the technical quality of the digital health technology, and its convenience and cost.

#### How Does Digital Health Technology Support Family Caregivers of Patients With Cognitive Impairment?

Although family caregivers believe that their involvement in the therapy provided to their relative leads to a better relationship, they may require more support from, and collaboration with, health care providers. The success of digital health technology implementation seems to be influenced by family caregivers’ participation as active facilitators in the delivery of care, the ease of use of digital health technology, access to the internet, and the caregivers’ literacy and technology literacy.

## Discussion

### Principal Findings

This scoping review aimed to portray the use of digital health technology to assist health care professionals and family caregivers caring for patients with cognitive impairment who live in the community or in a facility. Literature on the topic is scarce, recent, and heterogeneous. Previous literature [39] explored the use of eHealth technologies as a plausible approach to supporting aging with cognitive impairment and identified cognitive training solutions and supportive web platforms as the most effective interventions on a limited number of outcomes. More research is needed to solve methodological difficulties observed in the current literature, according to Dequanter et al [39], and development should focus on solutions for leisure and reminiscence as well as outcomes directly relevant to independent living. Di Lorito et al [40] gathered evidence on the effectiveness of digital health interventions on physical, cognitive, behavioral, and psychological outcomes as well as activities of daily living in people with dementia and mild cognitive impairment. The authors recommend considering different modalities of supervision while administering digital health interventions. “A mix of remote and face-to-face delivery could maximize benefits and optimize costs,” according to Di Lorito et al [40]. This paper is the first literature review that gathers the available digital health technology to support health care professionals and family caregivers caring for patients with cognitive impairment and synthesizes the implementation outcomes of using these technologies in the community or in a facility. We were able to retrieve only 7 manuscripts, including 6 (86%) peer-reviewed journal articles and 1 (14%) doctoral thesis, reporting on studies conducted over the last decade involving various technologies. Of these 7 studies, 4 (57%) reported on systems embedded into computers and tablet devices as gaming systems, self-management systems, and apps, whereas 3 (43%) reported on digital calendar reminder systems to support activities of daily living and medication management and a social robot used in therapeutic sessions. The interactions among patients with cognitive impairment, health care providers, and family caregivers identified in the included manuscripts were diverse and heterogeneous. Overall, the literature reported on the use of digital health technology to support bilateral interactions between patients and health care providers or between patients and family caregivers or a trilateral integration with support provided to all 3 groups, whereas 14% (1/7) of the studies reported on technology to support family caregivers only (people with dementia indirectly). The trilateral integration of technology complements the portrait presented by Huisman et al [41], who reported on technology targeting a person with dementia, the informal caregiver, or both.

Our scoping review indicates that the retrieved publications lack clarity regarding adopting a behavior change theory and associated model to carry out digital health technology intervention. Most (6/7, 86%) of the studies examined in this review did not include any explicit behavior change theory or model. The study by Cruz-Sandoval et al [34] was the only one reporting on using a behavior change theory, namely a comprehensive process model of engagement followed by cognitive stimulation therapy [34]. Two authors involved other theories such as activity theory [37] and simulated presence therapy intervention [35], but these are not behavior change models. The rest (4/7, 57%) of the articles did not mention any behavior change theory or model. This phenomenon can be explained by a lack of application of theoretical frameworks to conceptualize and govern the behavior change models that incorporate technology for patients with cognitive impairment. The shortcomings of using general theories and models for designing interventions aimed at this profile of patients with cognitive impairment were discussed in a recent scoping review of behavior change theories in adults without dementia [42]. The study aimed to adapt and develop a model promoting physical activity in people with dementia (the “PHYT in dementia”) [42] and presented an ad hoc model for the promotion of physical activity. Our scoping review indicates the direction that future studies may follow to eventually observe outcomes with a larger impact, that is, crafting a model that can be applied and replicated to inform the design of systems that support people with dementia to change their behavior.

Digital health technology can take multiple forms depending on the intervention it should deliver. It is important to note that only 14% (1/7) of the studies reported on implementing a robot as a conversational agent [34], and no other study presented another type of conversational agent, which can provide exciting opportunities for developing new ways of communicating with people with cognitive impairment. As other researchers have stated [43], implementing conversational agents to deliver interventions to people with dementia is still a poorly explored domain that deserves more attention and interdisciplinary work. This knowledge gap creates a new quandary in using established theoretical frameworks (eg, behavior change theories in our case) when building and deploying digital health technology. Our findings show that the use of digital health technology may bring real-world evidence [34] that is not supported by existing theoretical frameworks. This gap in real-world evidence documentation is a whole understudied research question. One can wonder whether technology must be used to support the adoption of behavior change approaches or whether it is introducing a new strategy that may prove effective but has not yet been defined and theorized. This dilemma may also raise the fundamental question of the necessity for behavior change theories to assist health care professionals and family caregivers caring for patients with cognitive impairment. Digital health technology may be perceived as a novel technique that has shown effectiveness (real-world proof) but has yet to be conceptualized as a posteriori. In this context, Bell et al [28] emphasize the importance of including perspectives of young people and lived experience, motivational frameworks, and behavior change interventions in developing digital health solutions. Our scoping review revealed 2 critical factors to consider when comprehending digital health technology for patients with cognitive impairment. The first factor is the operation mode. Digital health technology can be designed to operate with, or independently of, other hardware or software. A technology that operates independently of other technologies is called a stand-alone technology, which is usually easier to learn and use than a non–stand-alone technology. The second factor is the number of users. Although some of the digital health technologies presented in the retrieved studies are considered stand-alone technologies, the interventions needed more support from others. Understandably, end users may need the support of other people around them, especially when the end users are patients with cognitive impairment. Often, digital health technology is designed to enable family caregivers to access it, too, even if they are not required to act to deliver the intervention.

The design of technology and the inherent intervention seems critical in implementing and using digital health technology with end users. Di Lorito et al [40] recommend considering accessibility, acceptability, and sustainability for end users as prerequisites for the development of digital health interventions. Beyond exploring the effects of digital health technology on patients with cognitive impairment and their interaction with health care providers and family caregivers, this scoping review aimed to identify what determines the success of a digital health intervention. Design wise, only 29% (2/7) of the studies included in this review followed a user-centered design approach, despite the importance of such methodology when addressing a specific population with particular needs, namely patients with cognitive impairment [21-23]. A secondary observation from our scoping review is that designing a digital health technology for a patient with cognitive impairment necessitates collaboration with secondary stakeholders such as family caregivers and tertiary stakeholders from specialized institutions and clinics, in addition to actively and repeatedly involving the end user. Hung et al [35] followed a thorough user-centered approach involving patients and caregivers using video-ethnographic methods, video-recorded observations, and health care professionals’ interviews. Andreassen et al [33] validated the app with end users [44] and caregivers [45], but there is no explicit design process described. Including caregivers in the design is uncommon (in this scoping review, only a single study [35] did so) and represents a future research possibility. It is also important to note the complete absence of co-design and cocreation of solutions when this has been demonstrated to be a valuable methodology [46]. Co-design can be challenging for people with severe dementia or cognitive impairments, but it is still viable for people with less complex conditions as well as caregivers [47,48]. Because of their firsthand experience, caregivers can contribute to cocreating solutions for contexts they know well. This type of information is difficult to capture, formalize, and convey in interviews but is more naturally communicated and incorporated in cocreation endeavors.

### Strengths and Limitations

The findings of this study contribute to filling a significant knowledge gap regarding the value of using emerging digital health technologies to assist health care professionals and family caregivers in caring for patients with cognitive impairment. Although the portrait is limited to 7 manuscripts, this scoping review presents many compiled results and recommendations based on the primary authors’ observations. This knowledge is one of the priorities identified in the literature over the last decade, and its importance has grown since the COVID-19 pandemic significantly affected the delivery of rehabilitation services. This paper has presented the evidence as of April 2021, and there may be ongoing yet unpublished research work applied to the COVID-19 context. Although the findings are helpful for pandemic-related decision-making, the presented findings were interpreted without regard for the pandemic. This review did not focus on addressing cognitive impairment from a therapeutic perspective, and we did not discuss the use of digital health technology as a clinical approach. Although it was built as exhaustive as possible, our search strategy may have lacked some terms; for example, we did not include critical MeSH terms such as *Mobile Applications* and *Telemedicine* because these were expected to yield the same manuscripts as search strings 1 to 6 (Multimedia Appendix 1). In addition, we planned to include only peer-reviewed articles, but we added a doctoral thesis because of the scarcity of articles. Therefore, we discourage hasty generalization of the results owing to the heterogeneity of the technologies and contexts of the interventions and the small number of manuscripts included. 

### Conclusions

This paper showed that despite a plethora of theoretical frameworks available on the use of behavior change models, there is an apparent lack of application of theories incorporating technology for patients with cognitive impairment. This scoping review depicted existing digital health technology to support health care providers and family caregivers while caring for patients with cognitive impairment and highlighted the lack of reliance on behavior change theories and models despite studies showing their positive effects on patients with cognitive impairment, health care providers, and family caregivers with no adverse effects on any digital health technology users. This scoping review depicted the benefits, challenges, and influencers of using digital health technology to support patients with cognitive impairment, health care providers, and family caregivers to bolster future research and implementation.

## Appendix

Multimedia Appendix 1 Search strategy.

## Abbreviations

MeSH: Medical Subject Headings
PRISMA-ScR: Preferred Reporting Items for Systematic Reviews and Meta-Analyses extension for Scoping Reviews
