# Supplementary material for: Digital Health Technology to Support Health Care Professionals and Family Caregivers Caring for Patients With Cognitive Impairment: Scoping Review
Source: JMIR Ment Health. 2023 Jan 11;10:e40330. doi: 10.2196/40330 (PMC9878361; doi:10.2196/40330)
Supplement: Multimedia Appendix 1 [file mental_v10i1e40330_app1.docx]

**Appendix 1**

**Search Strategy**

Medline (Ovid) 1946-2021

1 exp Computer Communication Networks/ or exp Videoconferencing/ or Telecommunications/ or Artificial Intelligence/ or Human-robot interaction/ or Self-Help Devices/ or Sensory Aids/ or Monitoring, Ambulatory/ or Wearable Electronic Devices/ or exp Cell Phone/ or Electronic Mail/ or virtual reality/ or telemetry/is or robotics/ or wireless technology/ (186392)

2 (location-data or wearable$ or AI or artificial-intelligence or self-help-device? or service-robot? or (assistive adj1 (robot? or robotic? or technolog* or health or living))).tw,kw. (56928)

3 ((intelligent or smart) adj1 (home* or technolog* or sensor? or environment or robot?)).tw,kw. (1703)

4 (mobile-app* or mobile-phone* or GPS or geoposition* or cell-phone or cellular-phone or smartphone or phone or text messag* or SMS or electronic mail or email or e-mail or digital-automation or smart-tech* or voip or voice-over-IP or voiceover or videophone or viber or skype or google-hangout* or zoom or webex or facetime or whatsapp or ICT or iphone or ipad or tablet or webbased-tool? or web-based-tool? or webcam* or web cam* or robot*).tw,kw. (177173)

5 (((virtual or remote) adj1 (communicat$ or call? or calling or interaction$)) or (telepresen* or teleconference or tele-presen* or tele-conferenc*)).tw,kw. (1320)

6 or/1-5 [Telecommunications/Smart Tech] (370506)

7 health behavior/ or health risk behaviors/ or exp self-examination/ or sleep hygiene/ or exp "treatment adherence and compliance"/ (303884)

8 exp Exercise/ (205794)

9 exp Self-Control/ (3370)

10 (behavio?r adj1 (change* or maint* or theor* or intervention* or adjust* or modif* or shift or transform* or transition*)).tw,kw. (26983)

11 (activity engage* or attitude change* or ego-deplet* or self-control or self-regulat* or habit*).tw,kw. (208769)

12 or/7-11 [Behavoural Change] (713678)

13 exp Dementia/ (172467)

14 (dementia* or alzheimer's disease or cognitive impair*).tw,kw. (251729)

15 exp Intellectual Disability/ or Motor Skills Disorders/ or exp Disabled Persons/ (163489)

16 ((intellectual* or mental or mobilit* or physical*) adj1 (disab* or deficien* or develop* or retard*)).tw,kw. (66501)

17 (disabled person* or person with disabilit* or handicapp* or visually impair* or motor skill*).tw,kw. (25856)

18 or/13-17 [Dementia or Intellectual/Mobility Disability] (507543)

19 6 and 12 and 18 (831)

20 19 not ((exp infant/ or exp child/ or adolescent/) not exp adult/) (770)

21 limit 20 to english language (746)
